# Supplementary material for: A Hessian-based decomposition characterizes how performance in complex motor skills depends on individual strategy and variability
Source: PLoS One. 2021 Jun 30;16(6):e0253626. doi: 10.1371/journal.pone.0253626 (PMC8244910; doi:10.1371/journal.pone.0253626)
Supplement: S1 Appendix — (A.1) Relation to Muller & Sternad 2004; (A.2) Relation to Cohen & Sternad 2009 (B) Examples of individual distributions of release parameters in unconstrained 6D throwing (C) Score sensitivity (Hessian) and action variability (covariance) in unconstrained 6D throwing; (D) Coordinate invariance; (E) Non-zero Hessian’s eigenvalues in the presence of redundant actions. (PDF) [file pone.0253626.s001.pdf]

A Hessian-based decomposition characterizes how performance in complex motor skills depends on individual strategy and variability

Paolo Tommasino[1*], Antonella Maselli[1,2], Domenico Campolo[3], Francesco Lacquaniti[1,4], Andrea d'Avella[1,5*]

1 Laboratory of Neuromotor Physiology, IRCCS Fondazione Santa Lucia, Rome, Italy
2 Institute of Cognitive Sciences and Technologies, National Research Council, Rome, Italy
3 Synergy Lab, Robotics Research Centre, School of Mechanical and Aerospace Engineering, Nanyang Technological University, Singapore, Singapore.
4 Department of Systems Medicine and Center of Space Biomedicine, University of Rome Tor Vergata, Rome, Italy,
5 Department of Biomedical and Dental Sciences and Morphofunctional Imaging, University of Messina, Messina, Italy.

* corresponding authors
tommasinopaolo@gmail.com (PT), a.davella@hsantalucia.it (AdA)

S1 Appendix

A. Relation to TNC approaches

A.1 Relation to Müller & Sternad 2004

In this section we show that, when the score function is smooth, and the action distribution is sufficiently localized, it is possible to derive (Hessian-based) analytic expressions to isolate the three components of the TNC approach by Müller and Sternad (1), shown in Fig.1. Given two experimental strategies, such as S_A and S_B in the figure, the TNC method requires the generation of surrogate data-sets, S_A^0, S_B^0 and S_A^{sh}, to decompose the difference in expected score $\Delta\bar{\pi} = \bar{\pi}(S_B) - \bar{\pi}(S_A)$, into the sum of four independent components: ΔC_1, or *covariation*, is the difference in expected score between the strategy S_A and the (surrogate) strategy S_A^0, that is obtained by removing (via random permutations) any linear/non-linear correlation between the variables of the data-set S_A. Similarly, for S_B, a surrogate uncorrelated data-set S_B^0 is used to quantify the difference in performance ΔC_2 due to covariations in S_B. Notice that S_A^0 and S_B^0 have the same mean ($\bar{\boldsymbol{a}}$ and $\bar{\boldsymbol{b}}$, respectively) as their original data-sets and only differ with respect to their original data-sets in terms of variability. A third surrogate data-set S_A^{sh} is generated by *shifting* the location of S_A^0 (i.e. $\bar{\boldsymbol{a}}$) to the average location $\bar{\boldsymbol{b}}$ of the S_B data-set. The *tolerance* component is hence quantified as $\Delta T = \bar{\pi}(S_A^{sh}) - \bar{\pi}(S_A^0)$, has the two data-sets have same variability and differ only in terms of their average location. Lastly, the *noise* component is extracted as the difference in average performance between the surrogate data-set S_B^0 and S_A^{sh}, i.e. $\Delta N = \bar{\pi}(S_B^0) - \bar{\pi}(S_A^{sh})$.

Assuming that motor strategies are drawn from a localized distributions, $S_A = \{\bar{\boldsymbol{a}}; \Sigma_A\}$ and $S_B = \{\bar{\boldsymbol{b}}; \Sigma_B\}$, our method allows the estimation of all four components without using surrogate data-sets and random permutation. In this case, the covariance matrices Σ_{A_0} and Σ_{B_0} of the uncorrelated strategies S_A^0 and S_B^0, can be simply computed as diag(Σ_A) and diag(Σ_B), i.e. as the matrix of the diagonal elements (variances) of Σ_A and Σ_B, respectively. Knowing the Hessian matrix $H_{\bar{\boldsymbol{a}}}$ and $H_{\bar{\boldsymbol{b}}}$ at the two locations $\bar{\boldsymbol{a}}$ and $\bar{\boldsymbol{b}}$, respectively, allows to approximate ΔC_1,ΔT, ΔN and ΔC_2, simply as:

$$\Delta C_1 = \bar{\pi}(S_A^0) - \bar{\pi}(S_A) \approx \left[\alpha(\bar{\boldsymbol{a}}) + \frac{1}{2}trace(H_{\bar{\boldsymbol{a}}}\Sigma_{A_0})\right] - \left[\alpha(\bar{\boldsymbol{a}}) + \frac{1}{2}trace(H_{\bar{\boldsymbol{a}}}\Sigma_A)\right] = \\ = \frac{1}{2}trace[H_{\bar{\boldsymbol{a}}}(\Sigma_{A_0} - \Sigma_A)]$$

$$\Delta T = \bar{\pi}(S_A^{sh}) - \bar{\pi}(S_A^0) \approx \left[\alpha(\bar{\boldsymbol{b}}) + \frac{1}{2}trace(H_{\bar{\boldsymbol{b}}}\Sigma_{A_0})\right] - \left[\alpha(\bar{\boldsymbol{a}}) + \frac{1}{2}trace((H_{\bar{\boldsymbol{a}}}\Sigma_{A_0})\right] = \\ = \alpha(\bar{\boldsymbol{b}}) - \alpha(\bar{\boldsymbol{a}}) + \frac{1}{2}trace[(H_{\bar{\boldsymbol{b}}} - H_{\bar{\boldsymbol{a}}})\Sigma_{A_0}]$$

$$\Delta N = \bar{\pi}(S_B^0) - \bar{\pi}(S_A^{sh}) \approx \left[\alpha(\bar{\boldsymbol{b}}) + \frac{1}{2}trace(H_{\bar{\boldsymbol{b}}}\Sigma_{B_0})\right] - \left[\alpha(\bar{\boldsymbol{b}}) + \frac{1}{2}trace(H_{\bar{\boldsymbol{b}}}\Sigma_{A_0})\right] = \\ = \frac{1}{2}trace[H_{\bar{\boldsymbol{b}}}(\Sigma_{B_0} - \Sigma_{A_0})]$$

$$\Delta C_2 = \bar{\pi}(S_B^{sh}) - \bar{\pi}(S_B) \approx \left[\alpha(\bar{\boldsymbol{b}}) + \frac{1}{2}trace(H_{\bar{\boldsymbol{b}}}\Sigma_{B_0})\right] - \left[\alpha(\bar{\boldsymbol{b}}) + \frac{1}{2}trace(H_{\bar{\boldsymbol{b}}}\Sigma_B)\right] = \\ = \frac{1}{2}trace[H_{\bar{\boldsymbol{b}}}(\Sigma_B - \Sigma_{B_0})]$$

Hence, it follows that the difference in expected performance between the two strategies can also be approximated as:

$$\Delta\bar{\pi} = \bar{\pi}(S_B) - \bar{\pi}(S_A) = \Delta C_1 + \Delta T + \Delta N + \Delta C_2 \approx \\ \approx \alpha(\bar{\boldsymbol{b}}) - \alpha(\bar{\boldsymbol{a}}) + \frac{1}{2}trace(H_{\bar{\boldsymbol{b}}}\Sigma_B) - \frac{1}{2}trace(H_{\bar{\boldsymbol{a}}}\Sigma_A) = \Delta\alpha + \Delta\beta \tag{1}$$

A.2 Relation to Cohen & Sternad 2009

An alternative TNC approach, the TNC-Cost, has been proposed by Cohen & Sternad (2) to overcome some of the limitations of the original TNC described in the Appendix A1. In the novel approach, the parameters of the decomposition are no longer dependent on the sequence of calculation, and the components are quantified with respect to an optimized data sets derived by transformation of one of the features.

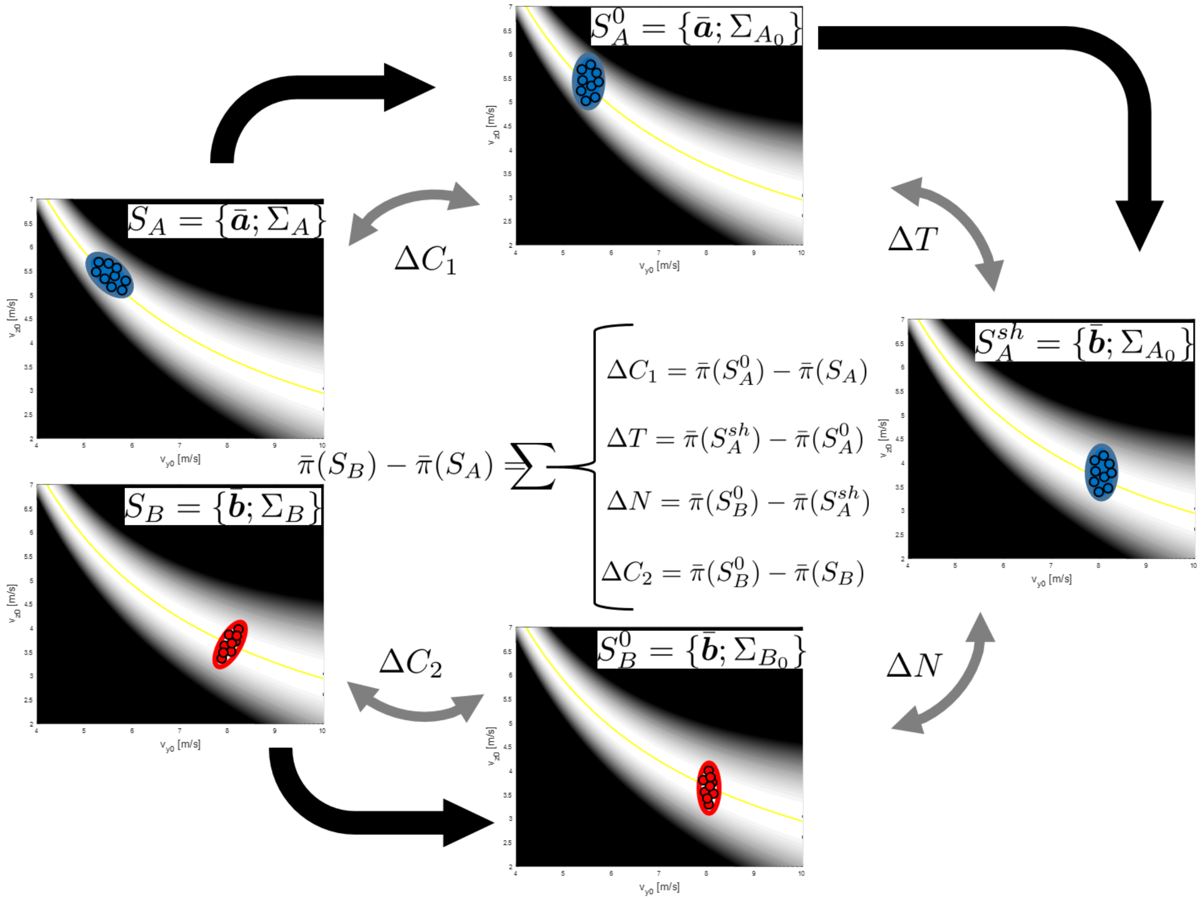

Figure 1. The TNC method proposed in (1). Given two datasets, A and B, for instance the release strategies of two different partecipants, the method requires the generation of surrogate data-sets S^0 (covariation-free) and S^{sh} (covariation-free but shifted mean). The difference in mean score between experimental and surrogate datasets are then used to calculate the relative tolerance ΔT, noise ΔN and covariation ΔC_1, ΔC_2 between the two strategies.

The T-Cost. The T-cost is the algebraic difference between the mean score of the experimental dataset and the mean score of an optimized dataset that differs from the first dataset only in terms of the mean action. More specifically, the action space is firstly discretized in a grid of n points that are used to shift the action distribution of the original dataset. Hence, for each grid point the original and the shifted dataset have the same action variability and only differs with respect to the mean action. For each grid points the method evaluates the mean score of each shifted dataset, such that the optimal one is the one which results in the minimal (i.e. best) mean score.

In the assumption that experimental dataset S_A has a small dispersion, we can apply our framework to calculate, analytically, the T-cost. In particular, let $\bar{\boldsymbol{a}}$ and Σ_A be the mean action and action covariance of the experimental dataset S_A, and let $\bar{\boldsymbol{a}}^*$ be the shifted mean of the

optimized data set S_A^*, then the T-Cost can approximated as:

$$\begin{aligned}\text{T-cost} = \bar{\pi}(S_A) - \bar{\pi}(S_A^*) &\approx \left[\alpha(\bar{\boldsymbol{a}}) + \frac{1}{2} trace(H_{\bar{\boldsymbol{a}}}\Sigma_A)\right] - \left[\alpha(\bar{\boldsymbol{a}}^*) + \frac{1}{2} trace(H_{\bar{\boldsymbol{a}}^*}\Sigma_A)\right] = \\ &= \alpha(\bar{\boldsymbol{a}}) - \alpha(\bar{\boldsymbol{a}}^*) - \frac{1}{2} trace\left[\left(H_{\bar{\boldsymbol{a}}} - H_{\bar{\boldsymbol{a}}^*}\right)\Sigma_A\right)\right] = \Delta\alpha + \Delta\beta.\end{aligned} \tag{2}$$

In the hypothesis that the original dataset had already an optimal mean action, i.e. on the solution manifold, and that the grid was sufficiently discretized such that also the optimal action belongs to the solution manifold, than the tolerance cost of Cohen & Sternad would correspond to a $\Delta\beta$ due to differences in the local geometry of the score between the experimental and the optimized datasets,i.e $H(\bar{\boldsymbol{a}}) \neq H(\bar{\boldsymbol{a}}^*)$.

Notice however, that the tolerance cost depends on the action covariance of the original dataset; hence it does not provide a unique measure of *tolerance/sensitivity* to errors of the score function. In other words, given two experimental datasets S_A and S_B with the same mean action $\bar{\boldsymbol{a}} = \bar{\boldsymbol{b}}$ but different covariances, one would measure a different Tolerance-cost for the two distributions, despite locally, the score has the same Hessians for both distributions,i.e. $H(\bar{\boldsymbol{a}}) = H(\bar{\boldsymbol{b}})$.

The N-Cost. The N-cost is the algebraic difference between the mean score of the experimental dataset and the mean score of a dataset with an optimized *noise*. More specifically, the optimal noise is obtained through a sequence of n steps which progressively shrunk the data points (the actions) of the original dataset towards its mean action. For each step, the mean score of the shrunk dataset is calculated and the optimal dataset is identified as the one with minimal mean score.

In the assumption that experimental dataset S_A has a small dispersion, we can apply our framework to calculate, analytically, the N-cost:

$$\begin{aligned}\text{N-cost} = \bar{\pi}(S_A) - \bar{\pi}(S_A^*) &\approx \left[\alpha(\bar{\boldsymbol{a}}) + \frac{1}{2} trace(H_{\bar{\boldsymbol{a}}}\Sigma_A)\right] - \left[\alpha(\bar{\boldsymbol{a}}^*) + \frac{1}{2} trace(H_{\bar{\boldsymbol{a}}^*}\Sigma_A^*)\right] = \\ &= \alpha(\bar{\boldsymbol{a}}) - \alpha(\bar{\boldsymbol{a}}^*) + \frac{1}{2}\left[trace\left(H_{\bar{\boldsymbol{a}}}\Sigma_A\right) - trace\left(H_{\bar{\boldsymbol{a}}^*}\Sigma_{A^*}\right)\right] \\ &= \frac{1}{2}\left[trace\left(H_{\bar{\boldsymbol{a}}}\left(\Sigma_A - \Sigma_{A^*}\right)\right)\right]\end{aligned} \tag{3}$$

where in the last identity we have used the fact that the experimental and optimized data-set have the same mean action, hence the same α and Hessian. This result shows that in general the N-cost depends on both the local tolerance of the score and the difference in covariance between the experimental and the optimized data-set. In the particular case in which the shrinking procedure returns an optimal dataset which is just a single point (2), i.e. $\Sigma_{A^*} = 0$, the N-cost corresponds to our β, highlighting the fact that this parameter does not in general provides a clear description of *noise* (meant as action variability) given that it mixes all the contributions that are due to the action variability and the geometry of the score.

The C-Cost. The C-cost is the algebraic difference between the mean score of the experimental dataset and the mean score of an optimized dataset that differs from the first only in terms of correlation between action variables, hence having the same mean action and uncorrelated variability of the original dataset. Following our framework, in the hypothesis of localized

distribution and smooth score function, the C-cost, or the difference between the original and the optimized dataset becomes:

$$\begin{aligned}\text{C-cost} = \bar{\pi}(S_A) - \bar{\pi}(S_A^*) \approx \left[\alpha(\bar{\boldsymbol{a}}) + \frac{1}{2}trace(H_{\bar{\boldsymbol{a}}}\Sigma_A)\right] - \left[\alpha(\bar{\boldsymbol{a}}^*) + \frac{1}{2}trace(H_{\bar{\boldsymbol{a}}^*}\Sigma_A)\right] = \\ = \frac{1}{2}trace\left[H_{\bar{\boldsymbol{a}}}\left(\Sigma_A - \Sigma_{A^*}\right)\right] = \frac{1}{2}trace\left(H_{\bar{\boldsymbol{a}}}T\right)\end{aligned} \tag{4}$$

where the T matrix has all zeros on the main diagonal given that the two datasets have the same uncorrelated noise.

B. Examples of individual distributions of release parameters in unconstrained 6D throwing

When actions are high-dimensional, as in unconstrained throwing where the action is a six-dimensional (6D) vector, it is impossible to visualize both the score and the individual strategies in a single plot, as in the 2D throwing case. Fig 2 shows the action score and individual throwing strategies in terms of pairs of release parameters (9 of the 15 possible pairs of 3 position and 3 velocity variables; *rows*) for five exemplary participants (*columns*) throwing at target T1. The last three *rows* are also illustrated in Fig 5 in the main text. Participants have been sorted from left to right according to their average release speed, hence $P10$ is the slowest thrower and $P11$ the fastest and they have similar horizontal and vertical release velocities as the five simulated 2D strategies illustrated in Fig 3B-C in the main text. The gray-scale shaded contours indicate the score associated to each pair of release parameters, i.e. the squared distance between the ball arrival position on the vertical target plane and the center of the target. The domain of each position and velocity variable corresponds to the population mean $\pm$ 3 standard deviations, while all the remaining release parameters are considered constant and fixed to the subject-specific mean action. There are 15 different gray-scale levels: the white area defines actions which have a score smaller or equal to 0.04 m^2, i.e. actions that land inside the target, which has radius 0.2 m. Notice that the regions corresponding to actions with the same score (i.e. same gray shading) have different shapes (or geometry) across planes and participants, as they depend on the individual mean release action. The wider the white areas around the mean release parameter, the more tolerant is the action-to-score function to stochastic perturbations. For instance, in the $v_{x_0} - v_{z_0}$ plane (*8-th row*), the lowest penalty (white) area looks like an ellipse, whose orientation suggests that the v_{z_0} direction is less tolerant/more sensitive to action variability and whose size, increasing with release speed, suggests that tolerance is higher for higher speed. In general, position variables appear more tolerant than velocity variables and the shapes of the white regions in the $p_{y_0} - p_{z_0}$ plane (*third row*), in the same-axis position-velocity planes (*fourth to sixth rows*), and in the $v_{y_0} - v_{z_0}$ plane (*ninth row*) indicate that release variables corresponding to the lowest score (throws hitting the target) are negatively correlated.

The distributions of the release parameters (*red circles*), summarized in each plot of Fig 2 in terms of mean (*blue circle*) and covariance (two-standard deviations, *blue ellipse*), also differs remarkably across planes and participants. For instance, $P11$ (*fifth column*), on average, releases the ball with a slower vertical velocity (v_{z_0}), compared to other participants, such as

$P10$ (*first column*) and $P1$ (*third column*), who instead throw with higher longitudinal velocity (v_{y_0}). The amount of variability in the $v_{x_0} - v_{z_0}$ (*eight row*) and $v_{y_0} - v_{z_0}$ (*ninth row*) planes also differs between the same three participants, as $P11$ shows a much wider covariance ellipses than $P1$ who, in turn, shows a wider covariance ellipses than $P10$. However, interestingly, their performance is similar, as the large variability of $P11$ in the $v_{x_0} - v_{z_0}$ and $v_{y_0} - v_{z_0}$ planes is partially compensated by being in a more tolerant region of the action score.

Also to notice are different patterns of covariance/correlation across individual strategies. For instance, $P10$, who is the least variable participant, shows no correlation between v_{y_0} and v_{z_0}, while $P11$, who is the most variable participant, shows a negative correlation: reducing the vertical release velocity proportionally to an increase in the longitudinal release velocity. This allows $P11$ to remain in the lowest penalty region as much as $P1$ and $P10$ and hence to have a similar mean score. Notice that $P15$ has a mean release velocity located close to the edge of the white region and hence on average does not hit the center of the target. However, $P15$ has a mean score similar to those of $P10$, $P1$, and $P11$, in part due to the small and, in most cases, well aligned covariance ellipses. Finally, $P18$, the best performing participant, shows mean release parameters at the center of all white regions and small and well aligned covariance ellipses.

In sum, the examination of the distribution of several pairs of release action parameters and the associated score suggests that individual throwing strategies differs in terms of mean action, action variability, and relationship between action variability and geometry of the action-to-score function. However, as the distribution is 6-dimensional and there are 15 different pairs of action variables, it is not possible to identify by visual inspection a unique source of the inter-individual differences in throwing strategies and to systematically explain the relationship between action distribution and throwing performance. These limitations can be overcome by introducing the Hessian-based decomposition of the mean score that we developed to provide a compact and informative description of the key features of the action distribution characterizing individual strategies and directly related to performance.

C. Score sensitivity (Hessian) and action variability (covariance) in unconstrained 6D throwing

The Hessian-based decomposition allows to characterize the structure of individual variability and its relation with the local geometry of the score, overcoming the limitations of a qualitative description of the action distribution and the score function, which is challenging when the score is defined over a high-dimensional space of action variables. Similar to the UCM and GEM method, which use Jacobian matrices to split motor variability along task-relevant and task-irrelevant directions, we use the Hessian matrix to quantify *score-relevant* variability affecting the mean score. Here, we illustrate how, across participants, a few eigenvectors of the Hessian matrix (principal sensitivity directions) associated with the largest eigenvalues identify the score-relevant directions that determine whether action variability affects the mean score or not. We characterize the structure of the Hessian and the action covariance matrices, which define the terms of the Hessian-based decomposition.

Fig 3A shows the distributions, across participants and for each target, of the eigenvalues or singular values of the Hessian matrix. Because in our throwing task the outcome space is two-dimensional, the solution manifold is a four-dimensional manifold embedded in the six-

dimensional action space (see Appendix C). Hence, on the solution manifold, the Hessian matrix will only have two non-zero singular values, whose associated eigenvectors or singular vectors defines a score-relevant plane. The corresponding eigenvalues quantify the sensitivity of the score function along that direction. Away from the solution manifold however, the Hessian matrix is also influenced by the non-linearities introduced by the mapping between actions and outcomes. Hence, for participants that do not have optimal mean actions, the Hessian matrix can have additional singular values which are different from zero. Fig 3A shows, however, that the contribution of the third and fourth singular value is negligible compared to the first two. Finally, focusing on the first two singular values, we notice that the sensitivity is slightly anisotropic, with the first sensitivity, on average, about 10% higher than the second. Furthermore, the first sensitivity shows the largest variability across participants and target conditions.

The two principal sensitivity directions $\boldsymbol{u}_1^H$, $\boldsymbol{u}_2^H$ define locally, i.e. around the mean action, a *sensitivity plane* embedded in the six-dimensional action space of the release parameters. Fig 3B shows the distributions of the principal sensitivity directions across participants and for each target. Across targets, the first principal sensitivity direction $\boldsymbol{u}_1^H$ is dominated by the vertical components (both position and velocity) of the release parameters and the second principal sensitivity direction is instead dominated by the lateral components (x_0, v_{x0}) of the release parameters.

In terms of action variability, for all participants and for all target conditions three principal components were able to explain 95% of the total variation, as shown in Fig 3C. Across participants and target conditions, the eigenvalue of the first principal component shows large variability across participants. It should be noted that the covariance matrix and, thus, the number of principal components is coordinate-dependent and in our scenario the action vectors contain both position and velocity variables which have different units. To assess the robustness of the estimation of the dimensionality of the action variability, we performed a principal component analysis on the correlation matrix rather than on the covariance matrix. The analysis of the eigenvalues of the correlation matrix confirmed that across participants and target conditions there were no more than three eigenvalues grater than one (3), supporting the conclusion that three components are sufficient to adequately describe the action variability.

Fig 3D illustrates the distribution of the first three principal variability directions $\boldsymbol{u}_1^\Sigma$, $\boldsymbol{u}_2^\Sigma$, $\boldsymbol{u}_3^\Sigma$, i.e. the eigenvectors of the covariance matrix associated to the three largest eigenvalues, indicating the directions along which most of the variation occurs. As for the principal sensitivity directions, a number of features of the principal variability directions are consistent across targets. For all four targets, the first direction captures a negative correlation between longitudinal (v_y) and vertical (v_z) release velocities and the second direction is dominated by the lateral velocity (v_x) but with variable contribution of the other velocity components. The distribution of all three directions, in larger measure for the second and third direction, however, are broader than those for the principal sensitivity directions, indicating that there are larger inter-individual differences in the structure of the action variability than in the sensitivity of the score around the mean action.

The distributions of principal sensitivity directions and principal variability directions illustrated in Fig 3B and D characterize features of the throwing strategy consistent across targets and participants. However, it is the selection of specific directions and, even more, their geometric relationship that determines the performance of individual participants. For example,

two strategies with identical mean action, which implies identical sensitivity, may have different mean scores because of different alignments of the principal variability directions with respect to the principal sensitivity directions. Fig 3E shows the absolute values of the scalar products between the 6 pairs of principal sensitivity directions ($\boldsymbol{u}_1^H$, $\boldsymbol{u}_2^H$) and principal variability directions ($\boldsymbol{u}_1^\Sigma$, $\boldsymbol{u}_2^\Sigma$, $\boldsymbol{u}_3^\Sigma$) in all 20 participants. Such complex pattern of scalar products between principal sensitivity and variability directions highlights a remarkable inter-individual variability in the relationship between action variability and score sensitivity.

D. Coordinate invariance

Approaches based on covariance matrices for the analysis of variability, such as the UCM, have often been criticized for their dependence on the choice of coordinates. Similar critiques have also been highlighted for the TNC approach that does not use (directly) covariance matrices: "for instance, one can always rotate the frame of reference to get variables that have zero covariance" (4). Furthermore, it is well known that Principal Component Analysis is sensitive to co-ordinates, especially when the multivariate data contains variables with different units. For instance, in this work the action vector contains position that are measured in meters and velocities that are measured in ms^{-1}. Should we rescale the action space to have comparable variances between positions and velocities? Would scaling affect our results? Here we show that this is not the case and that both ours and the TNC approach (1) are invariant under affine coordinate transformations. In fact, scaling, rotations and translations, i.e. any affine transformation of the action space, does not only affect covariance matrices (and hence correlations among variables) but also affects the performance manifold, in particular the structure of its Hessian.

Let's assume that we have two sets of coordinates $\{a\}$ and $\{b\}$ with which we can parameterize the n-dimensional action space $\mathcal{A} \subset \mathbb{R}^n$ and that the map $\boldsymbol{g}$ describe the relationship between the two coordinate system:

$$\boldsymbol{b} = \boldsymbol{g}(\boldsymbol{a}) \tag{5}$$

when the map is non-linear, its first-order approximation, around a point $\bar{\boldsymbol{a}}$ can be expressed as:

$$\boldsymbol{\delta}^b = J(\bar{\boldsymbol{a}})(\boldsymbol{\delta}^a) \tag{6}$$

where $J = \frac{\partial g}{\partial \boldsymbol{a}}$ is the $n \times n$ Jacobian matrix evaluated at $\bar{\boldsymbol{a}}$.

The score π is a scalar and therefore does not dependent on the choice of coordinates used to express the score function. In both coordinate systems we can write:

$$\pi = s^b(\boldsymbol{b}) = s^b(\boldsymbol{g}(\boldsymbol{a})) = s^a(\boldsymbol{a}) \tag{7}$$

By differentiating the last equality with respect to the $\{a\}$ co-ordinates, we find a well-known expression between the gradients in the two different co-ordinate systems:

$$\frac{\partial s^a}{\partial \boldsymbol{a}} = J^T \frac{\partial s^b}{\partial \boldsymbol{b}} \tag{8}$$

Differentiating again the above expression we can express the Hessian of the score in the two coordinate systems:

$$\frac{\partial^2 s^a}{\partial \boldsymbol{a}^2} = J^T \frac{\partial^2 s^b}{\partial \boldsymbol{b}^2} J + \left(\frac{\partial s^b}{\partial \boldsymbol{b}}\right)^T \frac{\partial J}{\partial \boldsymbol{a}} \tag{9}$$

Hence:

$$H^a = J^T H^b J + \frac{\partial J}{\partial \boldsymbol{a}}^T \boldsymbol{\nabla_b} s^b \tag{10}$$

When the average action belongs to the solution manifold ($\boldsymbol{\nabla_b} s^b = 0$), or when the change of co-ordinate is affine ($\frac{\partial J}{\partial \boldsymbol{a}}^T = 0$), the second term on the right hand-side is zero. In this case, the Hessian is a tensor and β does not depend on the co-ordinates used to parameterise the action space. In fact, let Σ^b, be the covariance of the action expressed in the $\{b\}$ coordinates, then, if the distribution is localized (small variability), the covariance in the $\{a\}$ co-ordinates can be estimated as:

$$\Sigma^a = J^{-1} \Sigma^b J^{-1^T} \tag{11}$$

and hence:

$$\beta = trace(\frac{1}{2} H^a \Sigma^a) = trace(\frac{1}{2} J^T H^b J J^{-1^T} \Sigma^b J) = trace(\frac{1}{2} H^b \Sigma^b) \tag{12}$$

where we have used the cyclic properties of the trace ($trace(ABC) = trace(CAB)$) to simplify the last equality.

Conversely, for highly non-linear change of coordinates, or for average actions that are 'far' from the solution manifold, the second term on the right-hand side of (10) may not be negligible. In such case, the Hessian looses its tensorial property, and β becomes a co-ordinate dependent measure:

$$\beta = trace(\frac{1}{2} H^a \Sigma^a) = trace(\frac{1}{2} H^b \Sigma^b) + trace\left(\frac{1}{2}\left(\boldsymbol{\nabla_b} s^b\right)^T \frac{\partial J}{\partial \boldsymbol{a}} \Sigma^b\right) \tag{13}$$

E. Non-zero Hessian's eigenvalues in the presence of redundant actions

In human motor control, the map between action and task variables represents a 'change of co-ordinates' $\boldsymbol{x} = \boldsymbol{f}(\boldsymbol{a})$, which often is non-linear and redundant. This latter properties of the map, makes the Jacobian $J = \frac{\partial \boldsymbol{f}}{\partial \boldsymbol{a}}$ a rectangular matrix with n columns (dimension of the action space) and m rows (dimension of the task space). In this case, equation (10) becomes:

$$H^a = J^T H^x J + \frac{\partial J}{\partial \boldsymbol{a}}^T \boldsymbol{\nabla_x} s^x \tag{14}$$

where H^x and $\boldsymbol{\nabla_x} s^x$ are the $m \times m$ Hessian and the $m \times 1$ gradient of the task-score function, respectively, and H^a is the $n \times n$ Hessian of the action-score function. Again, either on the solution manifold, or for a linear map between actions and outcomes, the second term on the right-hand side disappears, and H^a will only have $m < n$ non-zero eigenvalues. Conversely, away from the solution manifold and for highly non-linear change of co-ordinates, the term $\left(\boldsymbol{\nabla_b} s^b\right)^T \frac{\partial J}{\partial \boldsymbol{a}}$ will in general affect the number of non-zero eigenvalues, as well as the symmetry and positive-definiteness of the Hessian matrix.

References

1. Müller H, Sternad D. Decomposition of Variability in the Execution of Goal-Oriented Tasks: Three Components of Skill Improvement. Journal of Experimental Psychology: Human Perception and Performance. 2004;30(1):212–233. doi:10.1037/0096-1523.30.1.212.

2. Cohen RG, Sternad D. Variability in motor learning: relocating, channeling and reducing noise. Exp Brain Res. 2009;193(1):69–83. doi:10.1007/s00221-008-1596-1.

3. Kaiser HF. The Application of Electronic Computers to Factor Analysis. Educational and Psychological Measurement. 1960;20(1):141–151. doi:10.1177/001316446002000116.

4. Smeets JBJ, Louw S. The contribution of covariation to skill improvement is an ambiguous measure: Comment on Müller and Sternad (2004). Journal of Experimental Psychology: Human Perception and Performance. 2007;33(1):246–249. doi:10.1037/0096-1523.33.1.246.

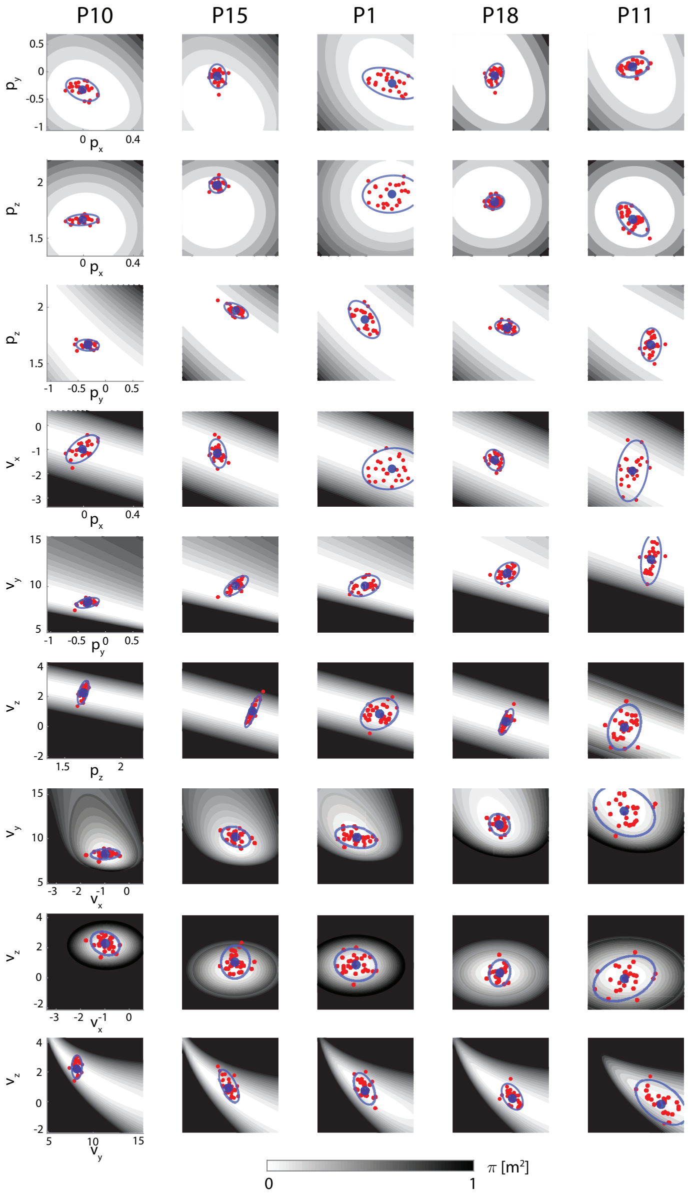

Figure 2. Examples of distribution of release position and velocity for five representative participants (target T1). Each row illustrates a pairs of release parameters, including all combinations of position variables (p_x-p_y, p_x-p_z, p_y-p_z), all combinations of position and velocity variables for each axis (p_x-v_x, p_y-v_y, p_z-v_z), and all combinations of velocity variables (v_x-v_y, v_x-v_z, v_y-v_z). Red circles represent release parameters of individual throws. Blue circles and ellipses represent mean and covariance (two standard deviations) of each parameter distribution. The gray level map shows the local score, as a function of the release parameters, underlying each throwing strategy. Note that differences in the background geometry of the maps reflects individual differences in the average action (mean position and velocity vectors at release). The wider the white area around the mean action, the more tolerant the score is to stochastic perturbations. Participants have been sorted from left to right according to their average release speed.

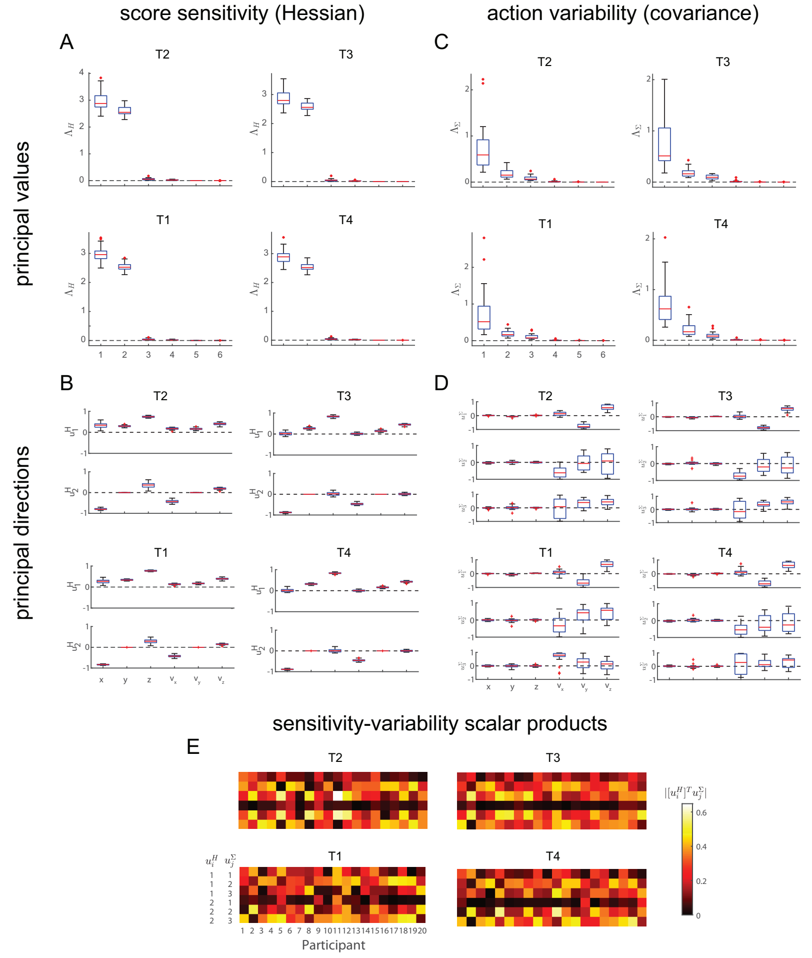

Figure 3. Sensitivity of the action-to-score function (Hessian matrix), action variability (covariance matrix), and their relationship across participants. (A) Distributions of the Hessian eigenvalues across participants for each target. In our scenario, the outcome space is two-dimensional and the solution manifold is a four-dimensional manifold embedded in the six-dimensional action space (see Appendix S1 Appendix C). Hence, the local tolerance of each participant is dominated by the first two eigenvalues of the Hessian matrix. (B) Distributions of the first ($\boldsymbol{u}_1^H$) and second ($\boldsymbol{u}_2^H$) principal sensitivity directions across participants for each target. The first principal sensitivity direction is dominated by the vertical release position and velocities while, the lateral and longitudinal components contributes 'equally' for target T1 and target T2, while for target T3 and target T4, the longitudinal components were 'more score relevant' than the lateral ones. The second principal sensitivity direction is instead dominated by the lateral release position and velocity. (C) Distributions of the eigenvalues of the covariance matrix across participants for each target. The first three principal components explain 95% of the total variation. (D) Distributions of the first ($\boldsymbol{u}_1^H$), second ($\boldsymbol{u}_2^H$), and third ($\boldsymbol{u}_2^H$) principal variability directions across participants for each target. (E) Absolute values of the scalar products between all pairs (i, j) of the first two principal sensitivity directions ($\boldsymbol{u}_i^H$) and the first three principal variability directions ($\boldsymbol{u}_i^\Sigma$).
