# Supplementary material for: A Hessian-based decomposition characterizes how performance in complex motor skills depends on individual strategy and variability
Source: PLoS One. 2021 Jun 30;16(6):e0253626. doi: 10.1371/journal.pone.0253626 (PMC8244910; doi:10.1371/journal.pone.0253626)
Supplement: Attachment — Submitted filename: Responses to editor and reviewers.pdf [file pone.0253626.s003.pdf]

**Editor (*PLOS Computational Biology*)**

*The reviewers remained positive about the potential of the Hessian-based decomposition method and appreciated the careful attention to their previous comments and the extensive work that has gone into revising and improving the manuscript. The exposition in the current version is improved. The paper successfully introduces the new approach and demonstrates how it contrasts with existing approaches. Unfortunately, however, as highlighted by Reviewer 2, the paper still has some significant shortcomings. In particular, to meet the standard for PLOS Computational Biology, we require that a paper should "demonstrate both methodological and scientific novelty, and provide profound new biological insights" (see our Scope: https://journals.plos.org/ploscompbiol/s/journal-information) and, while the novelty is evident, it's not clear the new method has convincingly demonstrated any clear new insights about behavior that would not be apparent by using other methods. What the new method does do very effectively is provide a compact characterization of how a given pattern of variability will interact with task geometry to determine performance. However, in the end what this yields is simply another way of describing the behavior and, while this seems to a promising domain in which to visualize the data - particularly for high dimensional tasks - it does not seem to reveal much structure beyond that induced by the task itself; it doesn't provide any specific insights into why participants should converge on the particular patterns of variability that they do, or why different participants converge on different solutions.*

While the Editor of *PLOS Computational Biology* has acknowledged the novelty and effectiveness of our method, the main criticism, and the reason for rejecting the manuscript (after two rounds of revisions), concerns the lack of "any clear new insight about behavior that would not be apparent by using other methods". We believe such criticism is not relevant when considering the suitability of our manuscript for publication in *PLOS ONE*, as original research methods are within its scope, according to the criteria for publication. Indeed, we have demonstrated the utility of our method, by showing that it provides a decomposition of performance into key indicators derived from the features of the action distribution of high-dimensional tasks, and we have validated it on high-dimensional experimental data from an unconstrained throwing task. However, we do believe that our method also provides new biological insights. In fact, the method reveals, through an analytical decomposition what are the key features of the action distribution affecting performance and it highlights the existence of two different types of performance trade-offs. Remarkably, our approach relies on a general mathematical formulation that applies to any behavior for which it is possible to associate a quantitative metric of goal attainment to the action performed to achieve such goal. Such insights cannot be derived neither from methods such as UCM and GEM, based on the Jacobian of the action-to-outcome mapping, because they only focus on outcome variability rather than performance over repeated actions, nor from TNC methods, because they cannot be applied to high-dimensional actions, as they are limited by their computational rather than analytical implementation. We do believe that additional insights will arise by using our method to characterize individual learning strategies, as it will provide a compact description of how individual strategies evolve during learning, which can facilitate the investigation of the learning processes and underlying neural mechanisms. However, this should not obscure the fact the decomposition itself does provide novel biological insights, as variability and redundancy are general features of any biological system performing goal-directed actions, and our method explains in a principled way how the interplay between variability, redundancy, and the geometric property of the action-to-outcome function of a specific task determines performance.

**Reviewer 1**

*Reviewer #1: In this revision the authors adequately addressed the reviewers' comments and significantly improved the manuscript. This revision presents a clear view of the method and the insights it enables, comparing to previous methods. The methods advantages are clearly illustrated through the simulation and the experimental data.*

We appreciate the fact that the reviewer acknowledges the insights and advantages provided by our method.

*Minor*

*At the start of the experimental result (section 2.2), it would be good to add a brief description of the experiment and the results. This is also where Fig1 should be.*

As suggested, we added a brief description of the experiments and the results at the start of section 2.2, including a reference to section 4.5 in the Methods where the details of the experimental setup and protocol are provided. Fig. 1 was already referenced at the beginning of section 2.2. We prefer to first refer to Fig. 1 in the Introduction, as we use introduce and exemplify a high-dimensional, redundant task and to highlight the large inter-individual performance differences typically observed in such tasks.

**Reviewer 2**

*The authors diligently responded to our long list of previous comments. Thank you, this was a lot of work! In response, the authors also have substantially reworked the manuscript, and it is indeed better now. Our concern about the correctness of the definition of theta (comment 2) has also been dealt with. Several superfluous results and figures have been removed from the main text. The pairwise comparison of the Hessian method with the three previous methods, TNC, UCM and GEM, is very informative about the differences between the four methods. In sum, the analytical method for the decomposition of variability via the Hessian is an excellent contribution to advance understanding of human movements, especially when the movements are complex and high-dimensional.*

We appreciate the reviewer's acknowledgment that our method contributes to the advancement of the understanding of complex and high-dimensional human movements.

*Yet, even after all these improvements, the manuscript remains a lot to digest. From my modest opinion, less would be more. Focus would make the manuscript more accessible and impactful. While sections were taken out and put into the appendix, some changes also added again more to the manuscript. The appendix is also very long and, from my perspective, unnecessary bulk for the present manuscript. In fact, some of the experimental data could be the starting point for a separate study with targeted analyses.*

We believe that the detailed comparisons with the TNC methods and the additional analyses of the experimental data, originally included in the main text and then moved to the Appendix in the R2 version of the manuscript, may be valuable for some of the readers. Including them in the Appendix is a reasonable compromise between the need for sufficient space to adequately introduce a novel method and its validation on experimental data and the need for a compact and focused

presentation. The fact that the Appendix is long should not be a problem for readers who are interested in understanding the key contribution of the paper, as the Appendix is not required to do so. Incidentally, the significance of some of the analyses presented in the Appendix had been highlighted by reviewer #1 of the original version of the manuscript, who even suggested to move part of the Appendix to the main text.

*The paper has two parts: The theoretical goal is to introduce a new promising analytical method for understanding variability in high-dimensional redundant motor tasks. This stage includes direct quantitative comparisons with similar methods highlighting the differences between the now four methods. The additional scientific question is to better understand inter-individual differences in human performance, a question that has long been overshadowed by the attempts to understand mean behavior.*

*The first part of the manuscript is excellent, the second part remains inconclusive. While the paper trimmed many of the confusing and lengthy descriptions of results, the application of the method to the experimental data are still largely inconclusive. The conclusion seems to be that "individual throwing strategies differ in terms of mean action, action variability, and relationship between action variability and geometry of the action-to-score function" (citation from appendix). While very plausible, this is scientifically not very satisfying. After the compression of high-dimensional spatiotemporal behavior into 5 indices, the only result is that the newly derived indices differ between subjects. While exemplary comparisons are made, these comparisons largely exemplify the method and do not help understanding the origin or meaning of individual styles. The manuscript has offered no real answer to the question of inter- individual differences. For example, does individual performance correlate with any of the 5 indices or combinations thereof? Do they remain invariant across practice, i.e. present the signature of a specific performance? Or, the most difficult but pressing question, can we learn anything about the underlying control?*

We appreciate the positive assessment of the first part of the manuscript (the introduction of the method and the comparison with existing methods using data from a simulated simplified throwing task) but we disagree on the statement that the second part (the application of the methods to experimental data from an unconstrained throwing task) is inconclusive.

First, our conclusion is that "the indicators derived from the Hessian-based decomposition allow to identify specific and consistent features relating individual throwing strategies to different performance level and to understand how different strategies achieve similar performances" (citation from the "Summary and conclusions"). The conclusion quoted by the reviewer from the Appendix, actually refers to the analysis of the examples of action distributions of the 15 pairs of action variables, and it is not the general conclusion of our work. Indeed, it refers to a descriptive characterization of the data that we introduced to highlight the need for a quantitative analysis. Thus, we agree that the description of the action distribution per se is not very satisfying. In fact, in the remainder of the paragraph of the Appendix from which the reviewer excerpted the quotation, we stated: "However, as the distribution is 6-dimensional and there are 15 different pairs of action variables, it is not possible to identify by visual inspection a unique source of the inter-individual differences in throwing strategies and to systematically explain the relationship between action distribution and throwing performance. These limitations can be overcome by introducing the Hessian-based decomposition of the mean score that we developed to provide a compact and informative description of the key features of the action distribution characterizing individual strategies and directly related to performance."

Second, it is not true that our method does not indicate how "individual performance correlate with any of the 5 indices or combinations thereof", as stated by the reviewer. As clearly expressed by Eq. 1, individual performance correlates with the sum of alpha and beta.

Third, understanding the origin or meaning of individual styles and the underlying mechanisms are desirable ultimate aims of our research approach and they are clearly fundamental questions in motor neuroscience. However, we believe it is unfair to criticize our work simply because it does not provide an answer to all relevant questions in the field. On the contrary, we believe that our method provides a significant advancement towards answering such fundamental questions. It clarifies what are the key features relating individual action distributions and performance, additionally highlighting how these features can be combined in ways that do not affect performance (according to two possible trade-offs) and in ways that do affect performance (in the alpha-beta plane, along direction orthogonal to the alpha + beta = performance lines).

*The authors now included a direct quantitative comparison of the three previous methods. However, seeing these differences, the immediate question is: do we gain different insights from the four methods, or should some results be regarded incorrect, or more or less trustworthy? It would be good to add a comparison of the four methods on the same data set, i.e., take one simulated or experimental data set and apply the four methods to these data. Then, we can directly compare the different information gained. What are pitfalls? What is ground truth?*

The main insights gained from the comparison of the four methods on the 2D throwing simulated data are: 1) both UCM and GEM do not allow in general to discriminate strategies with different performances (e.g. strategies with different tolerance-variability are associated to the same UCM and ORT variance in Fig. 4B, and with the same goal-relevant sensitivity and goal-relevant variability in Fig. 4C), i.e., they do not explain what features of the action distribution relate to performance; 2) TNC-cost analysis does discriminate strategies with the same performance but it does not allow to properly interpret the contribution of the different features (e.g. in the example of Fig. 4A, strategy with the same amount of noise are associated to different N-costs) and does not allow to identify trade-offs between different features (as the different costs of the TNC-cost analysis do not provide a decomposition of performance).

It would be interesting to perform a comparison of our decomposition and the TNC-cost analysis using the 6D throwing experimental data. Unfortunately, the TNC-cost analysis cannot be practically applied to 6D data, as the computation of the T-cost requires an exhaustive search on a grid in action space, and the number of grid points increases exponentially with the number of dimensions. Taking for example 600 points in each dimension (as in Cohen and Sternad 2009), the search must be performed on $600^6 \approx 4.6 \times 10^{16}$ points. Similar considerations apply for the C-cost computation.

*The manuscript is evidently very technical, which is good. However, this should not obviate the need for a conceptual introduction of the concepts that are then mathematically defined. I fear that the manuscript does not draw in readers who are not thoroughly familiar with the three extant methods. The authors are evidently steeped into all the issues, forgetting where newcomers are. It may be good to consider, once again, whether the basic questions and concepts are sufficiently introduced. Do readers arrive at a thorough understanding of the 5 indices and how they are necessary to understand complex behaviors?*

We believe that in our latest extensive revision (R2) of the manuscript we had made a significant effort to clearly introduce the basic questions and concepts without too many technical details (by presenting only the essential elements of the decomposition in the Results and moving the Methods at the end of the manuscript) and to explain how the 5 indices are necessary to understand how specific features of the action distribution relate to performance (using the 2D throwing simulated data). Indeed, we had added Fig. 2 in R1, as suggested by the same reviewer, to introduce the necessary concepts and variables more clearly. The same reviewer, in their comments to R1, had remarked that "the method presentation has improved with additional figures to show the conceptual essence in additional to the analytical derivation". We are now surprised that the reviewer still finds the conceptual introduction of the concepts unsatisfactory.

*The current terminology is still confusing at times. A clear distinction of these terms would be very helpful. For example, 'tolerance' is sometimes used 'noise tolerance', the index is for 'tolerance-variability', 'noise' is sometimes 'uncorrelated noise', or sometimes used without relation to the methods. Other times, it refers to the component of the TNC method Noise-cost. It is also important to clearly distinguish between covariation, as the name in TNC, as a description for alignment, and covariance as the matrix terminology. Bias (alpha) and tolerance-variability – variability is a word on its own, so is tolerance. Example: line 444: "larger variability is compensated by lower alignment and higher tolerance" here tolerance is used by its own, variability is used as a single word, but previously was connected with action variability.*

Tolerance (tau) always refers to noise tolerance. We could repeat "noise tolerance" every time but we believe it is not necessary as it is not ambiguous.

Tolerance is also part of the "tolerance-variability index" as tolerance (tau) is mathematically one of its components (beta = theta / tau x eta). We do not really see any confusion between tolerance (a property of the action-to-score function, depending only on the mean action) and tolerance-variability index (an index that depends on the interplay between action-to-score function and action variability).

Noise (eta) has a simple and clear mathematical definition (the trace of the covariance matrix, often referred to as "total variation" in the literature) which also correspond to the intuitive notion of total amount of noise. To further enhance the clarity of the manuscript, we have revised the entire text removing any reference to "uncorrelated noise" and using only "noise", "amount of noise", or "total variation".

*The figures have improved a lot. I would only suggest to make the axis labels bigger. Sometimes the Greek symbols almost disappear in the numbers, e.g., in Figure 2 and 3.*

We have increased the size of the axis label Figures 3, 4, 7, 8 and increased the size of the Greek symbols in Fig. 2.
