# Supplementary material for: A Hessian-based decomposition characterizes how performance in complex motor skills depends on individual strategy and variability
Source: PLoS One. 2021 Jun 30;16(6):e0253626. doi: 10.1371/journal.pone.0253626 (PMC8244910; doi:10.1371/journal.pone.0253626)
Supplement: Peer review history

# Author comment: Author response to previous submission

**DOI:** 10.1371/journal.pone.0253626.r001

**Transfer Alert**

This paper was transferred from another journal. As a result, its full editorial history (including decision letters, peer reviews and author responses) may not be present.

1 Jun 2021

Attachment Submitted filename: Responses to editor and reviewers.pdf Click here for additional data file.

# Editor report: Decision Letter 0

**DOI:** 10.1371/journal.pone.0253626.r002

**Authors:** Welber Marinovic

10 Jun 2021

A Hessian-based decomposition characterizes how performance in complex motor skills depends on individual strategy and variability

PONE-D-21-18068

Dear Dr. d'Avella,

We’re pleased to inform you that your manuscript has been judged scientifically suitable for publication and will be formally accepted for publication once it meets all outstanding technical requirements.

Within one week, you’ll receive an e-mail detailing the required amendments. When these have been addressed, you’ll receive a formal acceptance letter and your manuscript will be scheduled for publication.

An invoice for payment will follow shortly after the formal acceptance. To ensure an efficient process, please log into Editorial Manager at http://www.editorialmanager.com/pone/, click the 'Update My Information' link at the top of the page, and double check that your user information is up-to-date. If you have any billing related questions, please contact our Author Billing department directly at authorbilling@plos.org.

If your institution or institutions have a press office, please notify them about your upcoming paper to help maximize its impact. If they’ll be preparing press materials, please inform our press team as soon as possible -- no later than 48 hours after receiving the formal acceptance. Your manuscript will remain under strict press embargo until 2 pm Eastern Time on the date of publication. For more information, please contact onepress@plos.org.

Kind regards,

Welber Marinovic

Academic Editor

PLOS ONE

Additional Editor Comments (optional):

The manuscript has been thoroughly reviewed by distinguished academics in the field and I share their positive assessments of the manuscript. It is a pleasure to accept your submission in Plos One. 

# Editor report: Acceptance letter

**DOI:** 10.1371/journal.pone.0253626.r003

**Authors:** Welber Marinovic

15 Jun 2021

PONE-D-21-18068 

A Hessian-based decomposition characterizes how performance in complex motor skills depends on individual strategy and variability 

Dear Dr. d'Avella:

I'm pleased to inform you that your manuscript has been deemed suitable for publication in PLOS ONE. Congratulations! Your manuscript is now with our production department. 

If your institution or institutions have a press office, please let them know about your upcoming paper now to help maximize its impact. If they'll be preparing press materials, please inform our press team within the next 48 hours. Your manuscript will remain under strict press embargo until 2 pm Eastern Time on the date of publication. For more information please contact onepress@plos.org.

If we can help with anything else, please email us at plosone@plos.org. 

Thank you for submitting your work to PLOS ONE and supporting open access. 

Kind regards, 

PLOS ONE Editorial Office Staff

on behalf of

Dr. Welber Marinovic 

Academic Editor

PLOS ONE
